# *Etlingera elatior* Extract promotes cell death in B16 melanoma cells via down-regulation of ERK and Akt signaling pathways

**DOI:** 10.1186/s12906-017-1921-y

**Published:** 2017-08-22

**Authors:** Aungkana Krajarng, Malin Chulasiri, Ramida Watanapokasin

**Affiliations:** 1 Chulabhorn International College of Medicine, Thammasat University, Bangkok, Pathumthani Thailand; 2 Faculty of Pharmacy, Mahidol University, Bangkok, Thailand; 3 Department of Biochemistry, Faculty of Medicine, Srinakharinwirot University, Bangkok, Thailand

**Keywords:** *Etlingera elatior*, B16 melanoma cells, Nuclear condensation, ERK, Akt

## Abstract

**Background:**

Torch ginger (*Etlingera elatior,* EE) is a ginger plant that found in Southeast Asia. Previous study showed its flowers and leaves composed of several flavonoids with anti-cancer activity. This study aims to investigate the mechanism of EE extract on cell death induction in melanoma cells.

**Methods:**

To carry out this study, the cytotoxic effect of EE extract was performed using MTT assay. Nuclear morphological change and loss of mitochondrial membrane potential were observed using Hoechst 33,342 and JC-1 staining. Flow cytometry using Annexin V/PI double staining assessed apoptosis, necrosis and viability. Caspase activity was detected by caspase activity kits. The expression of Bcl-2 family proteins, ERK and Akt signaling pathways were examined by Western blot analysis.

**Results:**

The treatment of EE extract resulted in a dose- and time-dependent reduction in cell viability in B16 cells. It also induced nuclear condensation, phosphatidylserine exposure, and loss of mitochondrial membrane potential, which are markers of apoptosis. Furthermore, the expression of Bim was increased instead of Bax and Bcl-2. The results also showed caspase-independent activity and the down-regulation of ERK and Akt signaling pathway.

**Conclusion:**

The results suggest that EE extract induced caspase-independent cell death via down-regulation of ERK and Akt pathways in B16 cells. This may be beneficial as a chemopreventive or chemotherapeutic agent in melanoma treatment.

## Background

Skin cancer is the most common cancer in the United States and Europe whereas is uncommon in Asia. The causes of skin cancer are from genetic mutation and also from environmental factors such as long time exposure to sunlight or UV light. Melanoma is less common cancer but is the most dangerous form of skin cancer because it can spread to other parts of the body. Melanoma is originated from the transformation of melanocyte, which produces the melanin pigment in the skin. Melanoma can occur anywhere on your body where there is melanin, even though it has not been exposed to sunlight [1]. However, the mechanism of melanoma is unknown. There are different types of currently used treatment for patients depend on the stage of melanoma. Mostly, surgery or wide excision is the major treatment and also combined with chemotherapy, radiation therapy or immunotherapy [2]. At present, herbal medicine, which is an alternative medicine, is becoming a new way for cancer treatment. Many clinical studies have shown a spectrum of anticancer activities of herbal medicine and used as a combination to improve the efficacy and decrease side effects of chemotherapy and radiation therapy [3].

Torch ginger, *Etlingera elatior* (EE), is a plant in the ginger family (Zingiberaceae). It is native in Thailand, Indonesia, Malaysia, and widely cultivated in Southeast Asia. Its flowers and leaves have been used as spices for food flavoring and as ornamentals. Furthermore, it is also traditionally used for treating earache and cleaning wound. Postpartum women boiled leaves of EE mixed with other aromatic herbs for bathing to remove body odour [4]. In Australia and Hawaii, it is cultivated for cut flower production [5]. The phytochemical screening of methanol extract of EE flowers showed the presence of flavonoids, terpenoids, saponins, anthocyanin and tannin [6]. The GC-MS results of flower extract showed the main components were 1-dodecanol, dodecanol, and 17-pentatriacontene [7]. It has been reported that leaves of EE composed of flavonoid including kaempferol and quercetin which showed high antioxidant activity and strongest tyrosinase inhibition activity [8]. Leave extract of EE also showed antibacterial activity against Gram-positive bacteria of *Bacillus cereus*, *Micrococcus luteus*, and *Staphylococcus aureus* but no activity on Gram-negative bacteria of *Escherichia coli*, *Pseudomonas aeruginosa*, and *Salmonella cholerasuis* [9]. It also exhibited antifungal activity on *Colletotrichum gloeosporioides* [10]. Moreover, EE has been shown anticancer activity against cervical cancer HeLa cells [11], breast cancer CEM-SS and MCF-7 cells [12] but non-cytotoxic effect to normal human liver WRL-68 cells and African green monkey kidney Vero cells [13]. Our preliminary study [14] has reported that EE extract could inhibit cell proliferation and could induce apoptosis by cell morphological changes and nuclear condensation in human epidermoid carcinoma. However, the mechanism of EE extract in melanoma is not clear.

Here, we show that EE extract induce caspase-independent cell death in mouse B16 melanoma cells via the inhibition of ERK1/2, p38 and Akt signaling pathway.

## Materials and methods

### Plant extraction

The flower extract of *Etlingera elatior* (EE) included 50% hydroglycol was obtained from Dr. Malin Chulasiri. The fresh flowers of EE were collected from Pathumthani province, Thailand in September 2013 and were authenticated by a botanist. A voucher specimen (number SJ 002) was deposited at the Faculty of Medicine, Srinakharinwirot University, Bangkok, Thailand for future reference. The fresh flowers of EE were dried at 50°C and then ground. The powdered of EE was macerated in 50% hydroglycol, which was prepared from proportion of water and glycols (propylene glycol or butylene glycol). After 3-day maceration, the EE extract was filtered through Whatman filter paper No.1. The filtrate was kept refrigerated until use.

### Cell culture

Mouse melanoma B16 cells and monkey kidney Vero cells were obtained from the American Type Culture Collection (ATCC, Manassas, VA). B16 and Vero cells were maintained as a monolayer in Dulbecco’s modified Eagle’s medium (DMEM, Gibco BRL, Grand Island, NY) supplemented with 10% FBS, 100 U/ml penicillin and 100 μg/ml streptomycin (GE Healthcare, Utah, USA) at 37 °C in a humidified atmosphere of 5% carbon dioxide (CO_2_). The medium was refreshed every 2–3 days. B16 and Vero cells were sub-cultured using 0.25% trypsin-EDTA when the cells reached about 70% confluence.

### Cell viability assay

Effect of EE extract on the cell viability was determined by using 3-(4,5- Dimethylthiazol-2-yl)-2,5diphenyl-2H-tetrazolium bromide (MTT) assay. B16 and Vero cells were seeded at a density 7 × 10^3^ cells/well in a 96-well plate and allowed to grow for 24 h. Cells were then treated with EE extract at various concentrations of 0, 5, 10, 15, 20 and 25 μg/ml while the control group was treated with 50% hydroglycol for 24 h. After incubation, 10 μl of 0.5 mg/ml MTT solution was added to each well and the plate was further incubated for 2 h at 37 °C. The water-insoluble formazan crystal was dissolved in DMSO and the absorbance was measured at 570 nm using a microplate reader (Multiskan EX, Thermo electron corporation, Finland). Cell viability was expressed as percentage of viable cells of treated cells to control cells. Cells were treated in triplicates and the experiments were repeated three times. The IC50 value was calculated using the software GraphPad Prism 3.03 (GraphPad Software, Inc., San Diego, CA, USA).

### Detection of nuclear morphology by Hoechst 33,342 staining

B16 cells were seeded at a density of 1.5 × 104 cells/well in a 48-well plate and allowed to grow for 24 h. The cells were then treated with 15 μg/ml of EE extract for 4, 8, 16 and 24 h. Then cells were stained with 5 μg/ml of Hoechst 33,342 (Molecular Probes, Invitrogen, USA) for 30 min. Stained cells were washed with PBS once and examined by using fluorescent microscope (Olympus, Tokyo, Japan).

### Detection of loss of mitochondrial membrane potential *(ΔΨm*) by JC-1 staining

To determine *ΔΨm*, the lipophilic cationic dye 5,5^′^,6,6^′^-tetra- chloro-1,1^′^,3,3^′^-tetraethylbenzimidazol-carbocyanine iodide (JC-1, Molecular Probes, Invitrogen, USA) was used. This dye concentrates as red aggregates in mitochondria that maintain high *ΔΨm*. Upon excitation at 480 nm, aggregates have a red emission light (590 nm) while monomers have a green emission light (525 nm). B16 cells were seeded at a density of 1.5 × 104 cells/well in a 48-well plate and allowed to grow for 24 h. The cells were then treated with 15 μg/ml of EE extract for 4, 8, 16 and 24 h. Then cells were stained with 10 μg/ml of JC-1 for 15 min. Stained cells were washed with PBS once and observed under fluorescence microscopy (Olympus, Tokyo, Japan).

### Phosphatidylesreine exposure

Phosphatidylserine exposure was detected using Dead Cell apoptosis Kit with Annexin V Alexa Fluor™ 488 &/Propidium Iodide (PI) (Molecular Probes, Eugene, OR). B16 cell were seeded in 6-well plates and treated with 15 μg/ml of EE extract for 0, 4, 8, 16, and 14 h. Cells were harvested and washed once with ice-cold PBS. Cell pellets were resuspended in 100 μl of binding buffer, 5 μl of Annexin V-FITC conjugate and 1 μl of PI was added. The cells were incubated at room temperature for 15 min in the dark. After adding 400 μl of binding buffer, the cells were analyzed using a FACScan flow cytometry (Becton Dickenson, San Jose, CA).

### Caspase activity

Caspase activity was detected using the caspase-Glo® 3/7, 8, and 9 assay kits (Promega, Madison, USA) according to the manufacturer’s instructions. Briefly, B16 cells were seeded into black wall clear bottom 96-well plate at the density of 1 × 104 cells/well in triplicate wells and cultured overnight for attachment. Then cells were treated with 15 μg/ml of EE extract for 4, 8, 16 and 24 h. After that 100 μl of caspase-Glo® reagents were added into each well. Samples were incubated for 30 min at room temperature and luminescence was measured using a microplate reader (Spark 20 M Multimode microplate reader, Tecan, Switzerland).

### Western blot analysis

B16 cells were seeded at 2 × 105 cells into a 35-mm dish and treated with 15 μg/mL of EE extract for several time points. Then, cells were harvested and lysed with RIPA lysis buffer (50 mM Tris–HCl, pH 7.5, 5 mM EDTA, 250 mM NaCl, 0.5% Triton X-100) containing 10 mM PMSF and Complete Mini Protease Inhibitor Cocktail (Roche Diagnostics GmbH, Mannheim, Germany). Proteins were separated by 10% SDS-PAGE and transferred onto polyvinylidene fluoride (PVDF) membranes (Pall Corporation, USA) for 1 h at 100 V with the use of a Mini Trans-Blot Cell® (Bio-Rad). After blocking in 5% bovine serum albumin (BSA), the membranes were incubated overnight at 4 °C with primary antibody (Cell Signaling Technology, Beverly, MA.) The membranes were washed in TBST (10 mM Tris, pH 7.5, 150 mM NaCl and 0.1% Tween 20) and the appropriate secondary antibody conjugated with horseradish peroxidase (Cell Signaling Technology, Beverly, MA) was added for 1 h at room temperature. Immunoreactive protein bands were detected by chemiluminescence using enhanced chemiluminescence reagent (ECL, Millipore, Bedford, MA).

### Statistic analysis

Reported data represent mean ± SD. All measurements were taken in triplicate from three independent experiments. Statistical significances between groups were analyzed using Student’s *t*-test and accepted at *p* < 0.05.

## Results

### Cytotoxic effect of EE extract

**Fig. 1:**
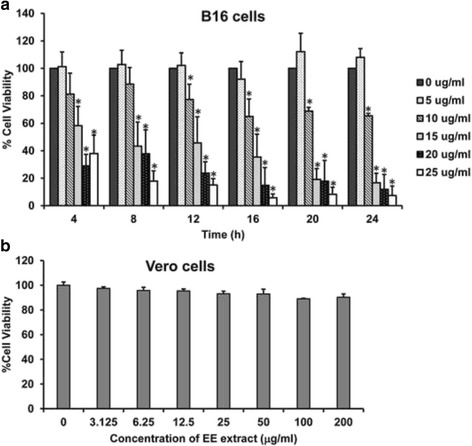
EE extract inhibited cell viability in B16 cells. The cytotoxic effect of EE extract was performed using the MTT assay. (**a**) B16 and (**b**) Vero cells were treated with various concentrations of EE extract at different time points for 24 h. Results are mean values ± SD of three independent experiments (*n* = 3). *p < 0.05 shows significant difference compared with control (0 μg/ml)

To investigate the cytotoxic effect of EE extract, the cancer cell line used in this study was mouse melanoma B16 cells whereas the normal cell line used was monkey kidney Vero cells. B16 and Vero cells were treated with various concentrations of EE extract for 24 h. Cell viability was assessed using MTT assay. The result revealed that EE extract decreased B16 cell viability in a dose- and time-dependent manner (Fig. 1a). In contrast, EE extract showed no cytotoxic activity against Vero cells (Fig. 1b). Accordingly, EE extract is only cytotoxic to melanoma cells and the IC_50_ of EE extract in B16 cells was about 15 μg/ml and this concentration was used in subsequent experiments.

### Effect of EE extract on nuclear morphological change

**Fig. 2:**
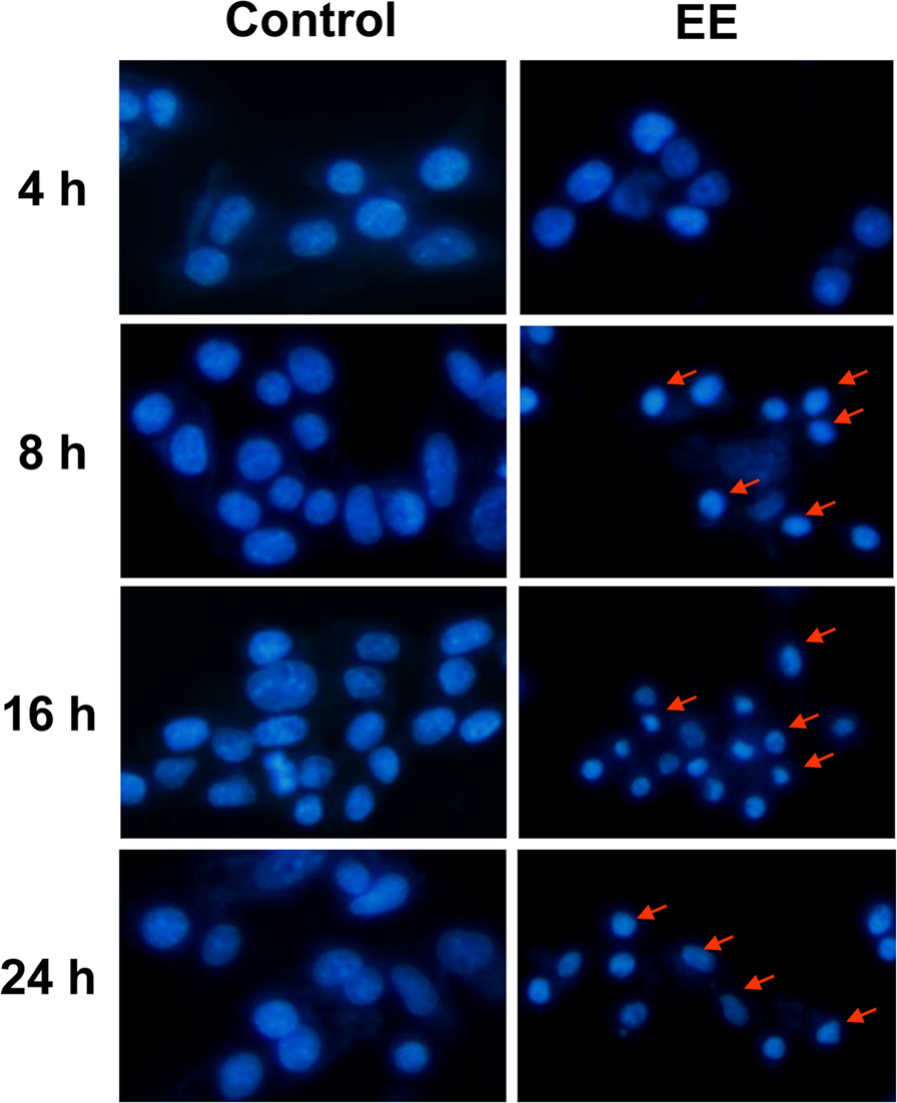
EE extract induced nuclear condensation in B16 cells. B16 non-treated or control (left panel) and treated with 15 μg/ml EE extract (right panel) for 24 h. Then cell were stained with Hoechst33342 and observed under a fluorescence microscope (20X magnification). The arrows show nuclear condensation of B16 treated cells

To investigate nuclear morphology of B16 cells treated with EE extract at several time points, cells were stained with Hoechst 33,342 and examined under fluorescence microscope. The result showed that EE extract could induce nuclear condensation but could not observe nuclear fragmentation (Fig. 2).

### Effect of EE extract on mitochondrial transmembrane potential (*ΔΨm*)

**Fig. 3:**
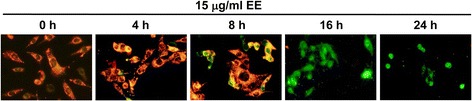
EE extract induced loss of mitochondrial membrane potential (ΔΨm). B16 cells were treated with 15 μg/ml EE extract for 24 h. Then cell were stained with JC-1 and observed at indicated time points under a fluorescence microscope (20X magnification)

Loss of mitochondrial membrane potential is one of characteristic in the induction of apoptosis. To measurement this event, B16 cells were stained with JC-1 dye. JC-1 is the unique fluorescent cationic dye that forms aggregates and emits a red fluorescence peak at high mitochondrial membrane potential, while at low mitochondrial membrane potential JC-1 exists as monomer form and emits a green fluorescence peak. Figure 3 shows progressive loss of red JC-1 aggregate fluorescence of the EE extract-treated cells, appearance of green monomer fluorescence in the cytoplasm at 8 h and complete loss of red fluorescence presence of only green fluorescence at 16 and 24 h. The changes from red to green fluorescence indicated a loss of mitochondrial membrane potential.

### Effect of EE extract on phosphatidylserine exposure

**Fig. 4:**
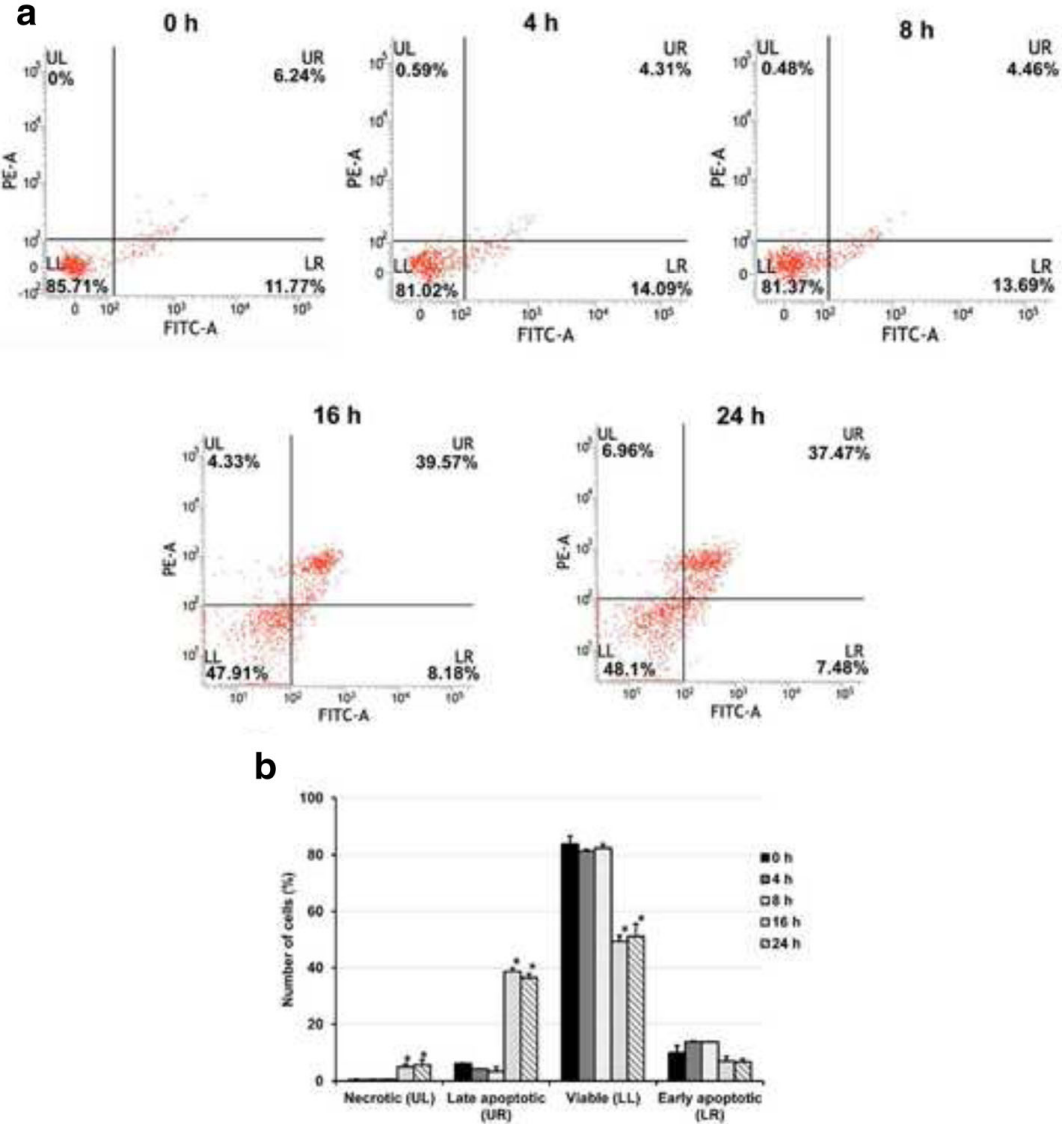
Effect of EE extract on phosphatidylserine exposeure for B16 cells. Cells were exposed to 15 μg/ml EE extract for 0, 4, 8, 16, and 24 h. (**a**) Representative dot plots of Annexin V/PI staining detected by flow cytometry are shown. The upper left (UL) quadrant contains the necrotic (Annexin V−/ PI+) population. The upper right (UR) quadrant contains the late apoptotic/necrotic (Annexin V+/PI+) population. The lower left (LL) quadrant contains the vital (double negative) population. The lower right (LR) quadrant contains the early apoptotic (Annexin V+/PI-) population. The result is from one experiment representative of three similar experiments. (**b**) The percentage of necrotic cells, early apoptotic cells, viable cells and late apoptotic cells at 0, 4, 8, 16, and 24 h are indicated. Results are mean values ± SD of three independent experiments (*n* = 3). *p < 0.05 shows significant difference compared with control (0 h)

Phosphatidylserine (PS) is a phospholipid which is a component of cell membrane. It indicates early apoptosis when PS is translocated from the inner to the outer leaflet of the cell membrane and display an “eat me” signal to prompt phagocytes to engulf the cell. Annexin V-PI double-labeling and FACS analysis were used for the detection of PS externalization. As shown in Fig. 4, the four quadrants in each panel correspond, respectively, to necrotic cells (upper left, UL), late apoptotic cells (upper right, UR), viable cells (lower left, LL), and early apoptotic cells (lower right, LR). Results showed that EE extract induced early apoptotic cells slightly about 14% at 4 and 8 h whereas late apoptotic cells increased highly about 40% at 16 and 24 h. These results suggest that EE extract induced phosphatidylserine exposure, a marker for apoptosis.

### Effect of EE extract on apoptosis induction

**Fig. 5:**
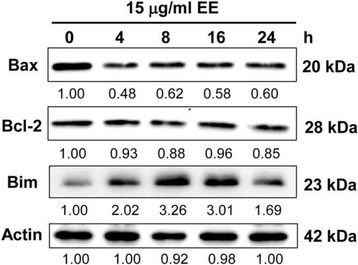
Effect of EE extract on expression of Bcl-2 family proteins in B16 cells. Cells were treated with 15 μg/ml EE extract for 24 h and examined by Western blot analysis. Actin was used as the internal control

The mechanisms of apoptosis can be divided into two major pathways; extrinsic or death receptor and intrinsic or mitochondrial pathways. Both pathways are activated by caspases. The Bcl-2 family of proteins, another mediator, has role as anti-apoptosis and pro-apoptosis. To observe whether EE extract induce apoptosis pathway, Bcl-2 family of proteins and caspase activity of caspase-3, −8, and −9 were detected. The results revealed no significant change in Bax and Bcl-2 levels were observed upon EE extract treatment. However, Bim, a pro-apoptotic BH-3 only member of Bcl-2 family, was increased compared with control group (Fig. 5).

**Fig. 6:**
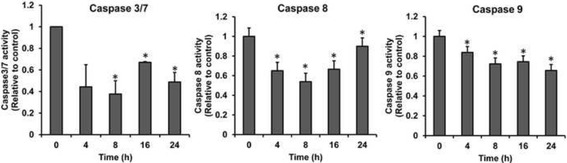
Effect of EE extract on activity of caspase-3/7, −8, and − 9. B16 cells were treated with 15 μg/ml EE extract for 24 h and detected caspase activity using the Caspase-Glo® kits. Data were normalized to the caspase activity of control and are shown as mean values ± SD of triplicate. *p < 0.05 shows significant difference compared with control (0 h)

Treatment of B16 cells with15 μg/ml EE extract at various times resulted in lower activities of caspase-3/7, caspase-8 and caspase-9 compared with control group (Fig. 6). Therefore, our results may suggest that EE extract induce cell death in melanoma B16 cells via a caspase-independent mechanism.

### Effect of EE extract on the expression of ERK and Akt signaling pathways

**Fig. 7:**
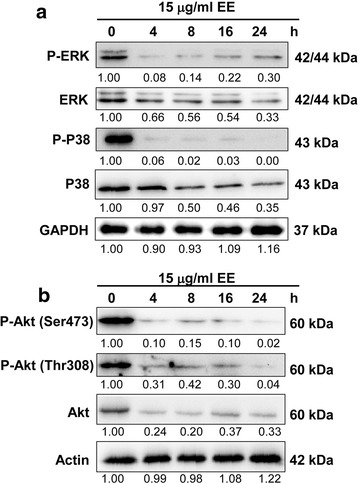
Effect of EE extract on expression of MAPK (**a**) and Akt (**b**) pathways in B16 cells. Cells were treated with 15 μg/ml EE extract for 24 h and examined by Western blot analysis. GAPDH and actin were used as the internal control

Mitogen-activated protein kinase or MAPK pathway is one of the drivers for melanoma. Members of the MAPK family are activated from extracellular signals via an ordered series of consecutive phosphorylation events, which associated with the regulation of cell survival or death. Extracellular signal-regulated kinase (ERK) promotes cell proliferation, differentiation and survival, whereas c-Jun N-terminal kinase (JNK) and p38 are known as stress-activated protein kinases. As shown in Fig. 7a, EE extract was able to decrease the level of phosphorylated and total ERK1/2 and p38 but unfortunately, both forms of JNK were undetectable.

AKT pathway is serine/threonine protein kinase that can promote cell survival, growth and proliferation. Phosphorylated Akt can inhibit the release of cytochrome c and apoptosis factor, thereby inhibiting apoptosis, and promote the growth of cancer cells. As seen in Fig. 7b, EE extract could decrease activity of total Akt and both forms of phosphorylated Akt after treatment for 4 h. Therefore, EE extract inhibited cell survival of melanoma B16 cells via Akt suppression.

## Discussion

It has been well known that dietary flavonoids, which commonly found in edible plants, have antioxidant effects, tumor cell growth inhibitory activity and apoptosis induction in cancer cell lines [15]. These could be served as chemopreventive agents. At present there are different forms of chemotherapy with several molecular targets but it is generally believed that chemotherapeutic agents kill cancer cells by induction of programmed cell death or apoptosis characterized morphologically by cell shrinkage, chromatin condensation and fragmentation and forming apoptotic bodies [16]. In this study, the results show EE extract, which composed of flavonoids, did not induce cytotoxic in normal Vero cells but could decrease cell survival of mouse melanoma B16 cells and also induce nuclear condensation. Remarkably, nuclear DNA fragmentation and apoptotic bodies were not found. Nevertheless, our results showed EE extract could induce phosphatidylserine exposure and loss of mitochondrial membrane potential using JC-1 staining, which are early markers of early phase of apoptosis.

On the other hand, Iwashita et al. [17] reported that several flavonoids including kaempherol and quercetin could inhibit the proliferation of B16 cells but both compounds seemed to have little effect in term of causing cell death. However, other flavonoids; isoliquiritigenin and butein, showed nuclear DNA fragmentation and apoptosis induction via the increase of Bax and suppression of Bcl-2 and Bcl-X_L_. Moreover, Zhang et al. [18] showed quercetin induced nuclear condensation and DNA fragmentation in a dose-dependent pattern in mouse melanoma B16 cells. The results also demonstrated quercetin led to loss of mitochondrial membrane potential and enhanced apoptosis by decreasing the expression of Bcl-2 and increasing of activity of caspase-3.

Two principle apoptosis pathways are the extrinsic and the intrinsic pathways. Each requires specific triggering signals to begin molecular events cascade regulated by the activation of caspases and the Bcl-2 family proteins [19]. The extrinsic pathway is triggered by death receptor-ligand binding such as Fas receptor and Fas ligand, then associated with the activation of caspase-8. The intrinsic pathway is triggered by cellular stress that results in release of cytochrome c from mitochondria into the cytosol and activation of caspase-9. Finally, both pathways initiate the activation of caspase-3 to induce cell death. The Bcl-2 family of proteins control mitochondrial membrane permeability and consist of pro-apoptotic such as Bax and Bak, and anti-apoptotic such as Bcl-2 and Bcl-X_L_ [20]. The third pro-apoptotic subgroup of Bcl-2 family of proteins is BH3-only protein such as Bim and Bid. Our results showed the level of Bim was increase whereas no effect in the level of Bax and Bcl-2 after EE extract treatment in B16 cells. In addition, EE extract did not induce any activity of caspase-3, −8, and −9. It may suggest that EE extract could induce apoptosis via caspase-independent and Bim expression. Similarly, Wang et al. [21] demonstrated that inhibition of MEK/ERK pathway induced caspase-independent apoptosis by the up-regulation of PUMA and Bim and down-regulation of Mcl-1 in human melanoma cells. Caspase-independent cell death pathway could be stimulated by factors released from mitochondria including apoptosis-inducing factor (AIF) and Endonuclease G (EndoG). Both factors translocate into nucleus and resulted in chromatin condensation and large scale (50 kb) DNA fragmentation [22]. The release of EndoG from mitochondria could be induced by BH3-only protein such as tBid and Bim and could be inhibited by Bcl-2 [23].

The regulation of Bim by ERK1/2 and protein kinase B (PKB) or Akt pathways is downstream of oncogenic protein kinases [24]. Oncogenes could inhibit or neutralize Bim which facilitate tumor cell survival. The oncogene-targeted therapeutics caused down-regulation of ERK1/2 and/or PKB signaling and promoted increased expression of Bim to induce cell death in tumor cells become interestingly [25].

Previous study showed melanoma cells were resistant to apoptosis induction via activation of ERK1/2 [26]. The histological results of Zhuang et al. [27] revealed high level of activated ERK1/2 (p-ERK) in primary melanoma. Therefore, activation of ERK1/2 may be important in the development and progression of melanoma. Pre-clinical and clinical trial studies developed the treatment of melanoma by inhibition of this signaling pathway target including using BRAF inhibitors; the upstream of ERK1/2 [28, 29]. Inhibition of ERK pathway in melanoma cells with BRAF inhibitors results in cell cycle arrest and promotes cell death including apoptosis. Enhanced inhibition of ERK pathway by combination treatment such as BRAF and MEK inhibitors could induce more cell death and plus with the change in tumor microenvironment may affect to tumor progression [30]. In addition, BRAF^V600E^ inhibitor PLX4720 inhibited ERK1/2 and induced apoptosis via Bim splicing in melanoma cells [31].

Ordinarily, Akt signaling pathway regulates diverse cellular functions including cell growth, proliferation, survival, and migration in tumor cells. Quercetin also inhibits the activity of many kinases including protein tyrosine and serine/threonine kinases [32, 33]. Previous study showed quercetin enhanced UVB-induced cell death in B16 melanoma cells. It markedly attenuated MEK-ERK signaling and also disrupted phosphatidylinositol-3-kinase (PI3K)/Akt survival signaling pathway [34]. This supported our results that EE extract down-regulated ERK and Akt signaling pathway in melanoma cells.

## Conclusion

The first choice for treatment of early melanoma is surgery, whereas the treatment with chemotherapy remains unsatisfactory because most patients were diagnosed at the late stage and they lost surgical opportunity. This study proposed that EE extract has ability to decrease cell viability of B16 melanoma cells and induce apoptosis through caspase-independent associated with down-regulation of ERK and Akt signaling pathways. This finding suggests that EE extract may be a chemopreventive or chemotherapeutic agent in skin cancer.

## Abbreviations

Akt: the serine/threonine kinase or protein kinase B (PKB)
Bak: Bcl-2 homologous antagonist killer
Bax: Bcl-2-associated death promoter
Bcl-2: B-cell lymphoma 2
Bcl-X_L_: B-cell lymphoma-extra large
BH3: Bcl-2 homology domain 3
Bid: BH3 interacting-domain death agonist
Bim: B-cell lymphoma interacting mediator of cell death
BRAF: v-raf murine sarcoma viral oncogene homologue B1
DMEM: Dulbecco’s modified Eagle’s medium
EE: *Etlingera elatior*
ERK: Extracellular signal-regulated kinase
FBS: Fetal bovine serum
h: hour
IC50: the half maximal inhibitory concentration
JC-1: 5,5^′^,6,6^′^-tetra- chloro-1,1^′^,3,3^′^-tetraethylbenzimidazol-carbocyanine iodide
JNK: C-Jun N-terminal kinase
MAPK: mitogen-activated protein kinase
Mcl-1: induced myeloid leukemia cell differentiation protein
min: minute
MTT: 3-(4,5-dimethylthiazol-2-yl)-2,5- diphenyl-2H-tetrazolium bromide
nm: nanometer
PUMA: p53 upregulated modulator of apoptosis
